# Developing Mineral Foam Blocks from Oil Shale Byproducts through Accelerated Carbonation

**DOI:** 10.1021/acsomega.5c05438

**Published:** 2025-10-02

**Authors:** Adheena Thomas, Can Rüstü Yörük, Mustafa Cem Usta, Nata-Ly Pantšenko, Tiina Hain, Mai Uibu, Andres Trikkel

**Affiliations:** † Department of Materials and Environmental Technology, 54561 Tallinn University of Technology, Tallinn 12616, Estonia; ‡ Department of Geology, 54561 Tallinn University of Technology, Tallinn 19086, Estonia; § Department of Civil Engineering and Architecture, 54561 Tallinn University of Technology, Tallinn 19086, Estonia

## Abstract

This study explores the impact of accelerated carbonation curing (ACC) on the production of sustainable mineral foam blocks (MFBs) for wall applications. MFBs were prepared with varying proportions of cement (CEM-I 42.5R) and oil shale ash (OSA), achieving 70–85% residual resource integration. Aluminum powder acted as a pore-forming agent to create the foamed structure. ACC conditions (100% CO_2_, 1 bar, ∼65% RH) enhanced performance metrics, which were evaluated by compressive strength, density, porosity, and CO_2_ uptake values. OSA incorporation can offer advantages in thermal properties of MFBs, yet without ACC treatment, the strength development of MFBs was primarily governed by cement hydration. Carbonated samples exhibited higher compressive strength (2.5–5.7 MPa) than uncarbonated ones (1.1–3.4 MPa). The analyses revealed partial carbonation of certain hydrated calcium-silicate and ettringite phases, while portlandite formed during hydration reactions was nearly fully converted to calcite. This conversion maximized CO_2_ sequestration (∼140 kg/ton), while maintaining a balanced strength. The role of anhydrite was found to be primarily *pH* dependent, participating in secondary reactions that enhanced the microstructure and integrity in conjunction with calcium silicate hydrates (C–S–H), calcium–silicate­(aluminum)-hydrate (C–S­(A)–H), ettringite, and gypsum. Mineralogical and microstructural analyses confirmed the formation of CaCO_3_ in various morphologies. Carbonation-induced densification, primarily driven by CaCO_3_ precipitation and transformation of amorphous phases, resulted in a more compact microstructure with reduced porosity. These findings demonstrate that ACC treatment improves MFB performance, optimizing high-volume OSA use while achieving significant CO_2_ mineralization, advancing sustainable construction.

## Graphical abstract

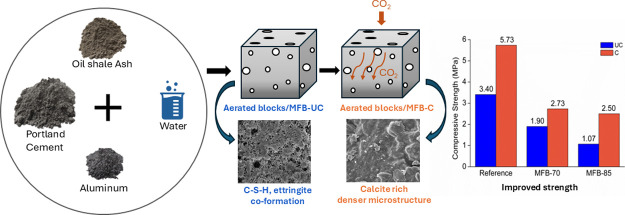

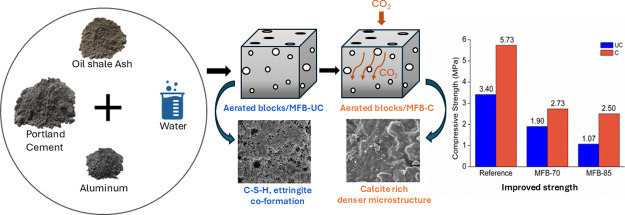

## Introduction

The use of industrial alkaline wastes as alternative binders or supplementary cementitious materials (SCMs) offers a dual advantage of mitigating waste disposal issues and reducing the carbon footprint of construction materials.^1–4^ Among various valorization strategies, accelerated carbonation curing (ACC) of CO_2_-reactive wastes enables simultaneous cementation and stabilization, contributing to circularity by enhancing mechanical performance and durability in cement-based systems.^5–8^ ACC treatment in freshly cast concrete enhances early age hydration, improving strength and durability by refining the microstructure and reducing porosity.^9,10^ This leads to greater resistance against permeability, chloride ingress, freeze–thaw damage, alkali-silica reaction, and sulfate attack.

Recent advancements in precast wall materials have promoted the use of inorganic, recyclable insulation solutions incorporating SCMs with ACC treatment. These advancements involve principles of circularity, particularly in nonautoclaved lightweight building materials,^11–17^ which are found to be less energy-intensive solutions. Porous materials such as cellular concrete, autoclaved aerated concrete, and mineral foam blocks (MFBs) offer enhanced sustainability and performance, including improved thermal and acoustic insulation. Their porous structure facilitates CO_2_ diffusion, enabling mechanical strengthening through CaCO_3_ precipitation during carbonation.

Common CO_2_-reactive industrial wastes used in such systems include calcium- and sulfur-rich materials like red mud, gypsum, metallurgical slags, fly ash, cement kiln dust, and oil shale ash (OSA).^18–21^ However, their variable mineralogy and presence of deleterious compounds (e.g., sulfates, alkalis, and organic carbon) can impair workability, increase water demand, and affect long-term performance, especially at high replacement levels.^22,23^ Additionally, SCM selection, blending ratios, water to binder ratio, and curing conditions remain critical for optimizing product performances. Since inadequate water retention—due to altered water to binder ratios or insufficient preconditioning—can cause unreacted minerals, pore blockage, and limited ion dissolution. Water loss from the exothermic carbonation process and untailored ACC treatment may impair strength development.^15–18^ These challenges underline the need for the careful selection and optimization of SCMs to tailor their properties for specific applications. Certain industrial wastes or ashes typically regarded as weak or neither with limited hydraulic nor pozzolanic properties (such as some containing calcium and magnesium silicates, portlandite, gypsum, etc.), can be activated with ACC treatment^24–30^ to obtain carbonation-induced solidification.

One such material with considerable potential for treatment through ACC is OSA, a calcium- and sulfur-rich byproduct generated from the combustion of oil shale. OSA can be used as a source material in composite cement production;^31^ however, despite the abundance of OSA generated from power production, its utilization remains quite low.^32^ Its direct application in cementitious systems has been limited due to its inherently low hydraulic and pozzolanic reactivity under conventional curing conditions. This low reactivity typically constrains the extent to which OSA can replace cement at high volumes.^33–35^ Importantly, the mineralogical composition of OSA closely resembles that of materials used in cellular concrete, which supports its suitability for activation via carbonation processes.^29,36–39^ Previous uses of OSA in lightweight building products—particularly those manufactured with autoclaving for thermal insulation—have achieved compressive strengths between 2.5 and 3.4 megapascals (MPa) and densities ranging from 600 to 700 kg per cubic meter.^36^

Estonia, home to one of the world’s richest oil shale reserves—alongside countries such as the USA, Jordan, and Brazil—still relies partially on oil shale for electricity generation and extensively on shale oil production. This has led to the accumulation of large quantities of OSA, which presents both environmental management issues and opportunities for material recovery and reuse.^39^ MFBs, when produced by using ACC treatment, can offer a promising route for the circular utilization of such industrial residues. The combination of ACC and MFB technologies may allow for the high-volume incorporation of OSA, helping to address environmental challenges while supporting the development of low-carbon construction materials.

This study investigates the potential for integrating high proportions of OSA—ranging from 70 to 85% by weight—into the production of MFBs using ACC treatment to improve its binding reactivity and performance. The MFBs were systematically evaluated for compressive strength, density, total porosity, and thermal conductivity, with specific attention given to the influence of carbonation curing. To elucidate mineralogical and microstructural transformations, advanced characterization techniques were employed, including Fourier transform infrared spectroscopy (FTIR), thermogravimetric analysis (TGA), X-ray diffraction (XRD), and scanning electron microscopy (SEM). Furthermore, the dissolution behavior of the materials was assessed by analyzing pH, electrical conductivity (EC), and the concentrations of Ca^2+^ and SO_4_^2–^ to gain insight into ion release and chemical interactions. Through this comprehensive investigation, the study aims to enhance the understanding of OSA-based mineral foams and their potential for environmentally sustainable construction applications.

## Materials, Sample Preparation, and Testing Methods

### Materials

In this study, Portland cement (PC) type CEM I 42.5 R, supplied by UNIKAKS AS, and aluminum powder (Al), serving as the aerating agent, were procured from Carl Roth GmbH and utilized in the experimental program. OSA, an industrial byproduct, was sourced from the Auvere Power Plant, which uses a circulating fluidized bed boiler for electricity generation. The plant primarily combusts oil shale, often cofired with up to 50% wood chips.^40^ OSA consisted of a blend of cyclone, filter, and electrostatic precipitator fly ashes.

#### Physical Characterization

For material characterization and sample preparation, the OSA was sieved through a 100 μm mesh to ensure a particle size distribution (PSD) comparable to that of the PC. The physical characterization of the PC, OSA, and Al powder included PSD and specific surface area (SSA) measurements (Table 1, Figure 1).

**Table 1:** BET SSA of PC and OSA and Cumulative Distribution of Particle Sizes at *d*_10_, *d*_50,_ and *d*_90_ of PC, OSA, and Al (nd: no data)

	*d*_10_ (μm)	*d*_50_ (μm)	*d*_90_ (μm)	SSA (cm^2^/g)
PC	6.56	14.96	39.37	2060
OSA	6.32	15.94	68.39	4340
Al	10.57	27.71	71.82	nd

**Figure 1:**
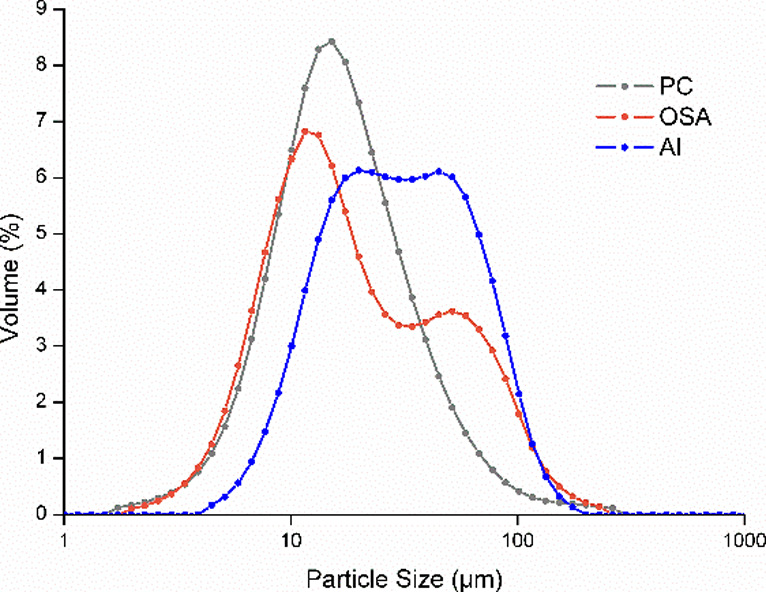
Particle size distribution of PC, OSA, and Al.

Horiba Laser Scattering instrument (LA-950 V2) was used for PSD measurement (with ethanol as a dispersant). The BET-N_2_ sorption method was used to measure the SSA with a Kelvin 1042 sorptometer.

#### Chemical Characterization

The chemical composition of PC and OSA was determined by XRF (BRUKER S4 PIONEER WDXRF, software: Eval) and is shown in Table 2.

**Table 2:** Chemical Compositions of PC and OSA

	PC	OSA
SiO_2_ (%)	19.47	7.21
TiO_2_ (%)	0.31	0.11
Al_2_O_3_ (%)	4.56	1.76
Fe_2_O_3_ (%)	3.07	2.63
MnO (%)	0.069	0.06
MgO (%)	3.56	3.55
CaO (%)	58.78	53.09
Na_2_O (%)	0.23	0.06
K_2_O (%)	1.06	0.18
P_2_O_5_ (%)	0.08	0.13
SO_3_ (%)	4.03	13.97
LOI (%)	3.96	16.63

Total carbon (TC) and total inorganic carbon (TIC) values were measured with an Eltra CS 580 Carbon Sulfur Determinator (Table 3), and total organic carbon (TOC) can be calculated as the difference between TC and TIC. The free CaO (*fCaO)* content was determined using the ethylene glycol method, which accounts for both CaO and portlandite (Ca­(OH)_2_) phases.

**Table 3:** TC, TIC, and TOC Values with *f*(CaO) of PC and OSA

	TC	TIC	TOC^a^	fCaO
PC	0.73	0.73	0	2.32
OSA	2.93	2.62	0.31	22.02

a TOC = TC-TIC.

The XRF analysis indicates that CaO, SO_3_, and SiO_2_ constitute approximately 75% of the major chemical components in OSA, while minor constituents include Al_2_O_3_, Fe_2_O_3_, TiO_2_, K_2_O, Na_2_O, MgO, and P_2_O_5_. Despite the high calcium content observed in both PC and OSA, as reflected by the CaO levels determined through XRF, the overall chemical and mineralogical compositions of the OSA and PC differ significantly. This distinction becomes more apparent when considering the loss on ignition (LOI), TC, TIC, and *fCaO* values. The LOI (16.63%) value and TIC (2.62%) content indicate that most of the carbon in the ash is in the form of mineral CO_2_, primarily within carbonates. The elevated concentration of *fCaO* (22.02%) in OSA highlights its potential for CO_2_ sequestration.

### Sample Preparation

Table 4 shows the experimental formulations of the prepared MFBs. There were two percentile variations: *MFB-70* (30%PC–70%OSA) with 0.6 water/solid ratio (w/s), and *MFB-85* (15%PC–85%OSA) with 0.625 w/s. A reference sample (*Reference-*100%PC with 0.5w/s) is included solely for comparative context, as well as to investigate the behavior of PC alone. This allowed for a better understanding of workability, product density, and the influence of cement on material performance.

**Table 4:** Experimental Formulations for Specimens

specimen	PC (%)	OSA (%)	Al (%)	w/s	spreadability (mm)
*Reference*	100		0.1	0.5	102
*MFB-70*	30	70	0.1	0.6	103
*MFB-85*	15	85	0.1	0.625	105

The amount of aeration agent was kept constant, i.e., 0.1 wt % Al powder was added to all the proportions. This value was obtained after a series of trials targeting common commercially available autoclaved aerated concrete density grades (500–700 kg/m^3^). For all batches, the w/s ratio of the slurry was fixed according to its flowability. The workability of slurry can be visually assessed by evaluating its viscosity. However, the commonly used slump test is unsuitable for low-density foam concrete.^41^ Instead, the spreadability method is recommended for determining workability.^42^ To measure the flowability of the mixes (excluding those containing Al powder), a custom-designed spread flow table was utilized. This apparatus consisted of a square metal plate and a cylindrical mold, securely held in place with a central handle and having a capacity of 60 cm^3^ (6 cl). The flowability of the mixes was optimized by adjusting their spread to a range of 100–105 mm, which was found to be ideal for casting. It was observed that flowability decreased with the incorporation of and the increase in OSA content. This reduction is likely due to the irregular morphology of OSA particles and higher SSA, which leads to higher cohesion and increased water absorption. The rapid reaction of CaO with water further contributes to the observed decrease in the flowability.

A schematic diagram illustrating the sample preparation and curing stages is presented in Figure 2. A kitchen blender (3000 rpm) was used for intensive mixing to guarantee a uniform mixture and slurry texture. OSA and PC were added to the blender and swirled for 1 min. After that, the required amount of distilled water, determined by the w/s ratio, and Al powder were added and mixed thoroughly for an additional 1 min. The slurry was poured into two-thirds of the 40 × 40 × 40 mm stainless steel molds without any vibration or compaction and kept in room conditions to avoid quick expansion and blasting of air bubbles during chemical expansion. The chemical expansion occurs due to the reaction of Al with calcium hydroxide and water, forming hydrated calcium aluminate and hydrogen gas (H_2_) (eq 1), which become trapped in the slurry, resulting in the formation of entrained air voids in the material.^43^

$$2\mathrm{Al}+3\mathrm{Ca}(\mathrm{OH}{)}_{2}+6H_{2}O\to 3\mathrm{CaO}\cdot {\mathrm{Al}}_{2}O_{3}\cdot 6H_{2}O+3H_{2}$$ 1

**Figure 2:**
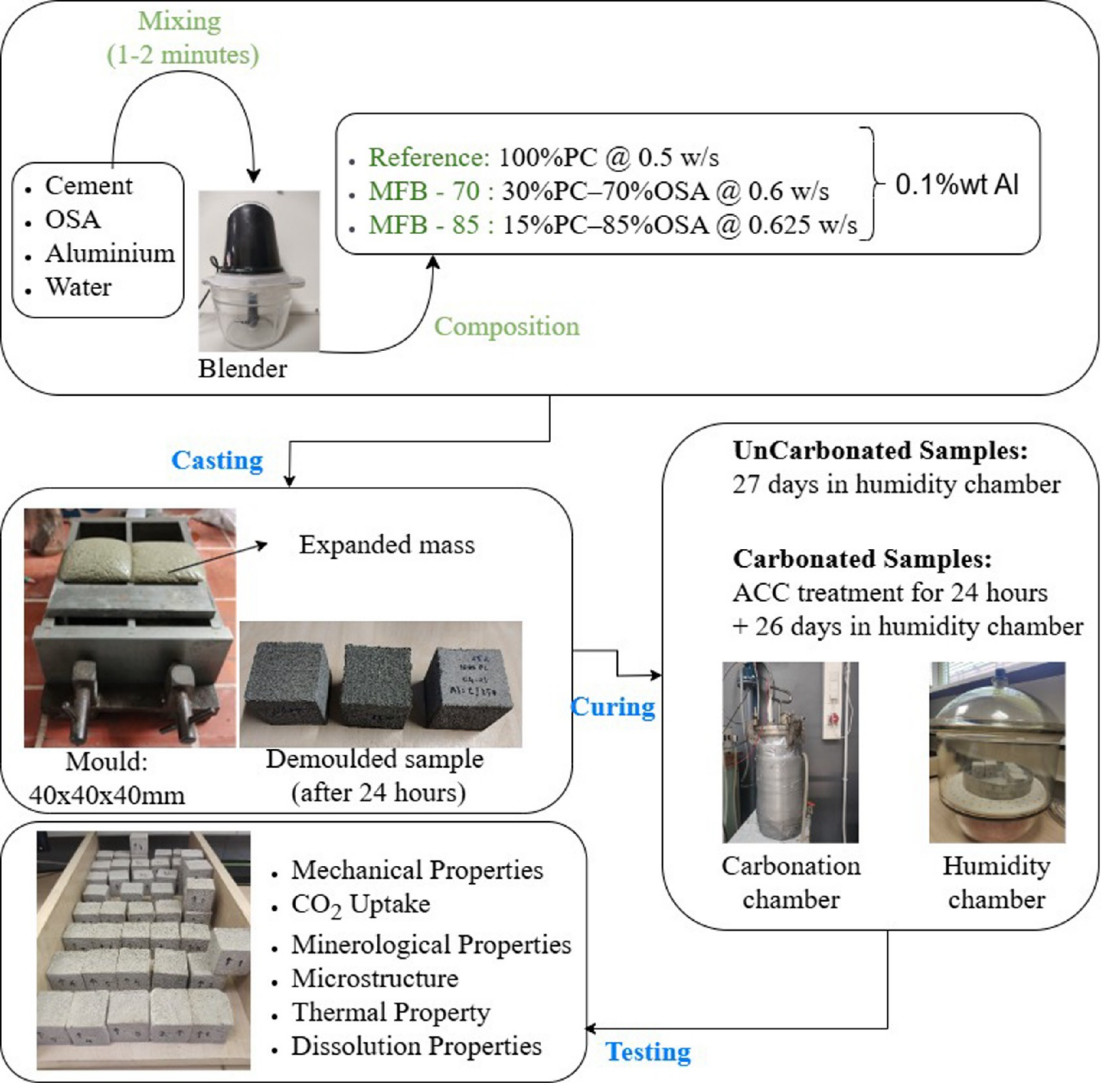
Schematic diagram representing the sample preparation and curing stages.

The expanded mass was cut from the top of the mold after six hours, and specimens were demolded after 24 h. Each set of specimens was divided into two groups: carbonated/ACC and uncarbonated. The carbonated group underwent ACC treatment after demolding for 24 h in a 100% CO_2_ environment at ambient temperature (∼65% relative humidity) and a pressure of 1 bar, before being moved to the desiccator for the remaining curing time (26 days). The uncarbonated group was placed in a desiccator right after demolding for 27 days to cure under controlled conditions (85% relative humidity and 22 ± 1 °C).

### Testing Methods

#### Compressive Strength, Density, and Total Porosity

Cubic samples with a side of 40 ± 0.5 mm were used for the compressive strength test. The tests were conducted using a compression testing machine according to EN 771-4:2021. Weight (weighing scale of accuracy 0.01g) and volume of samples were obtained to calculate their dry bulk density (ρb) after drying samples at 40 °C in a ventilated oven (24 h). The true density (ρt) was measured using a pycnometer (EN 196-6). The ρt is a measure of the density of a material excluding any pores or voids, making it essential for accurately assessing the total porosity (ε) of a sample. The total porosity is calculated using the following formulas

$$\varepsilon =\left(1-\frac{\rho b}{\rho t}\right)\times 100$$ 2

#### Thermal Properties (Thermal Conductivity, Diffusivity, and Heat Capacity)

EN 771–5:2011 requires thermal properties of manufactured stone masonry units to be measured per EBS-EN 1745:2020. However, sample sizes were incompatible with the heat flow meter apparatus. Instead, the EVS-EN ISO 22007-2:2022 (hot disc method), typically for plastics yet suitable for other materials, was applied, with the material’s properties within the standard’s range. Measurements were taken using a Hotdisk Thermal Constants Analyzer TPS2200, including a Keithley 2401 source and Keithley 2000 multimeter.

#### TGA-CO_2_ Uptake, FTIR and XRD

TGA was conducted to evaluate the thermal characterization of both carbonated and uncarbonated specimens, as well as to calculate the CO_2_ uptake, by using a Setaram Labsys 2000 thermo analyzer (10 K/min, sample mass: 25 ± 2 mg, 21%O_2_/79%Ar) with alumina crucible. To identify the mineralogical changes, FT-IR spectra was acquired using an FT-IR spectrometer (BRUKER, ALPHA) with platinum attenuated total reflectance (ATR and OPUS software was used. The mineralogical phase characterization of UC/C-MFB was further analyzed with the BRUKER D8 ADVANCE XRD using Topas and EVA software’s for data processing.

#### SEM

For SEM analysis, fragments of approximately 10 mm in size were obtained from specimens previously subjected to compressive strength testing using a hammer and blade. Owing to the uniform porosity and carbonation of the initial specimens, the selected fragments were considered representative of the bulk material. Prior to imaging, fragment edges were polished by using fine-grit sandpaper to ensure suitable surface quality. Later, the fragments were affixed to carbon adhesive tape on SEM stubs and coated with a gold–platinum alloy using a sputter coater for 100 s to enhance surface conductivity and minimize coating nonuniformity. Due to the high porosity of the samples, nitrogen gas was introduced during the vacuum process to stabilize the specimens. Microstructural characterization was performed by using a high-resolution Zeiss Merlin SEM equipped with an In-lens secondary electron detector for high-contrast surface imaging and an energy-selective backscattered electron detector for compositional contrast.

#### *pH*/*Ec* and Ion Leaching (Ca^2+^, SO_4_^2–^)

For the dissolution characteristics, suspensions were prepared according to EN 12457-2. The values were determined as the average of multiple measurements from different sets of samples, ensuring that the broken pieces used for testing were representative of both the inner and outer surfaces, even though the sample size was small.

For *pH* and *Ec* measurements, 4g of small broken pieces (broken after the compressive strength test) and 40 mL of distilled water were mixed in a centrifuging tube with a test tube rotator (GFL,3025, Germany) for 24 h. The mix is then kept in a centrifuge, and the suspension is vacuum-filtered (standard filter paper, pore diameter 0.45 μm). The *pH* values of the filtrate were measured using a Mettler Toledo SevenGo Duo Pro *pH*/Cond meter SG23. *Ec* values were measured using a Mettler Toledo InLab 738 ISM conductivity meter. Ca^2+^ and SO_4_^2–^ measurements were done by diluting the filtrate used for *pH* and *Ec* measurements at a suitable dilution factor. Ca^2+^ was determined using a SpectrAA 55B Flame Atomic Absorption Spectrometer, Varian, Inc., Palo Alto, CA, USA, and SO_4_^2–^ ions were determined with a Lovibond Spectro Direct spectrometer (methods: silver nitrate turbidity and barium sulfate turbidity).

## Results and Discussion

### Properties of the MFBs and Impacts of ACC

#### Density and Porosity

Earlier studies have shown that carbonation influences the porosity and density of cement-based materials,^44^ while OSA blending typically lowers density due to the porous nature and chemical composition of the ashes, which reduces the amount of denser cement in the mix.^45^ Similar trends are obtained while evaluating the dry density of MFBs. OSA blending reduces the density of the samples, resulting in ρb values of 601.3 kg/m^3^ for the *Reference*, 588.8 kg/m^3^ for *MFB-70*, and 574.1 kg/m^3^ for *MFB-85*, the lowest among them. In contrast, ACC treatment increases the density, with values rising to 698.7 kg/m^3^ for *Reference*, 685.7 kg/m^3^ for *MFB-70*, and 645.2 kg/m^3^ for *MFB-85*, which remains the lowest (Figure 3a). The ρt values, which correlate with ρb, clearly indicate that ACC treatment increases the material’s density due to the pore filling effects of CaCO_3_ precipitation^10,45,46^ (Figure 3b). It is known that the precipitation of CaCO_3_ can reduce capillary porosity, lowering the paste’s absorption and permeability in concrete, as well as conventional curing after carbonation can continue forming additional hydrates that grow and fill pores of carbonated-*MFBs*.^47,48^ The ε of the samples decreases after ACC treatment from 74.2–71.2% for the *Reference* sample (4%reduction), 73.7–71.6% (2.8% reduction) for *MFB-70,* and 74.6–72.7% for *MFB-85* (2.5% reduction) (Figure 3c), with *MFB-85* maintaining the highest porosity.

**Figure 3:**
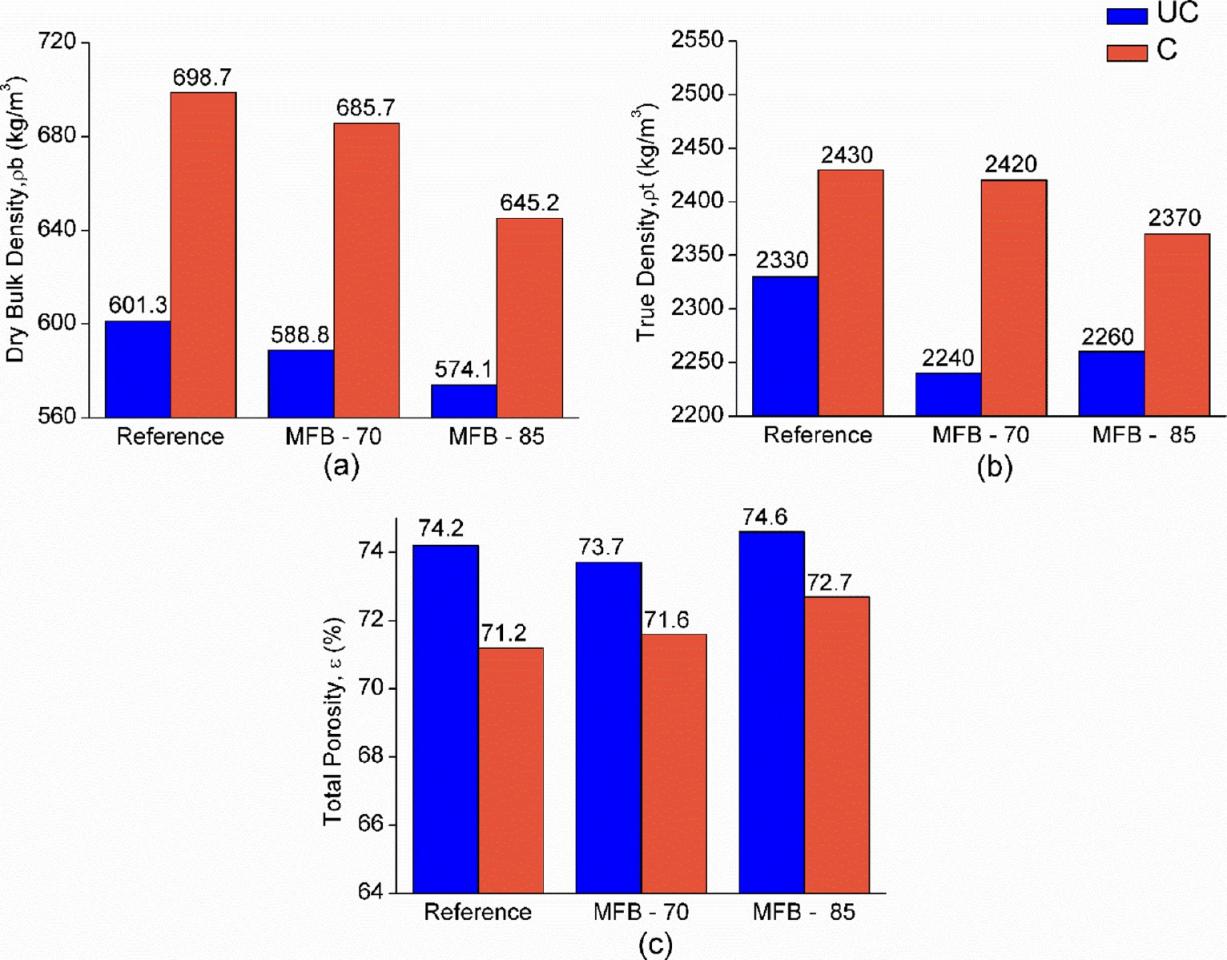
(a) Dry bulk density, (b) true density, and (c) total porosity of uncarbonated (UC) and carbonated (C) *Reference* and MFB specimens.

#### Compressive Strength

A direct relationship between density and compressive strength was observed in the MFBs, consistent with typical characteristics of cellular or autoclaved aerated blocks,^38^ where higher densities correlate with increased strength. The OSA/cement ratio significantly influences strength values in the absence of ACC treatment. Without ACC treatment, strength development is primarily governed by cement hydration, and OSA blending leads to a gradual reduction in strength. *MFB-70* exhibits 1.9 MPa, *MFB-85* the lowest at 1.07 MPa, while the *Reference* sample achieves the highest strength of 3.4 MPa. This decline in strength is largely attributed to the increased water demand due to the hydrophilic and porous nature of OSA, which results in a weaker early matrix and higher microporosity. Previous studies^49–51^ have also reported that the limited hydrous transformations occurring within various OSAs delay cement hydration and reduce hydration efficiency at higher blending ratios. These retardation effects are linked to a higher volume of micropores relative to the *Reference*, with hydration primarily involving *f*(CaO), anhydrite, secondary Ca­(Mg)-silicate minerals, and minor amorphous Al–Si glass phases forming secondary Ca-rich hydrates.^51,52^ These findings suggest that the incorporation of OSA (in conventional cement or sand replacing applications) can influence the material’s microstructure and hydration behavior, potentially leading to altered mechanical properties over time.

However, the complex interaction between the hydrous phases and carbonation processes highlights the role of secondary reactions associated with the CO_2_ mineralization, which should also be considered while determining the overall performance of OSA-blended cement systems.^50,53,54^

As shown in Figure 4, the ACC treatment significantly enhances the compressive strength of OSA-blended MFBs by promoting microstructural densification through carbonation reactions. The ACC treatment effectively compensates for the strength reductions typically associated with high OSA replacement levels. For example, the carbonated *MFB-70* exhibited a 40.5% increase in compressive strength, reaching values comparable to the *Reference*-UC sample, while *MFB-85* showed a 133% increase (2.5 MPa) relative to its uncarbonated counterpart. The *MFB-85* sample, due to its higher water-to-solid (w/s) ratio, initially formed a weaker and more porous matrix, making it more susceptible to microstructural densification via carbonation. This increased susceptibility may allow for greater CaCO_3_ precipitation, leading to a more refined pore structure. Consequently, the carbonation process has a more pronounced strengthening effect in high w/s systems than in denser, low w/s formulations.

**Figure 4:**
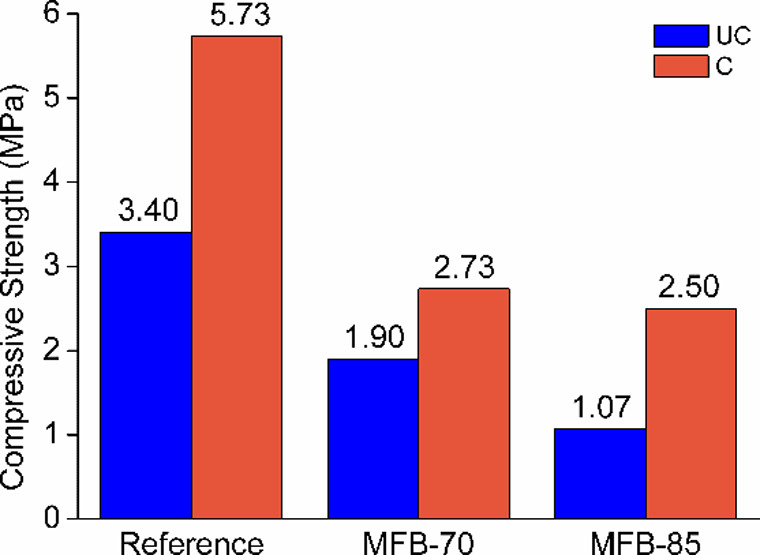
28 days compressive strength of uncarbonated (UC) and carbonated (C) *Reference* and MFB specimens.

#### Thermal Properties

In the context of building materials, lower thermal conductivity is highly desirable, as it minimizes heat transfer, enhances insulation performance, and reduces energy consumption. The thermal performance of MFBs was found to be closely influenced by the structural densities, including microstructural characteristics and their mineralogical composition (Figure 3a). The thermal conductivities (*k*) of carbonated-*Reference*, *MFB-70,* and *MFB-85* samples were measured as 0.21, 0.18, and 0.17 W/m·K, respectively (Table 5). The improvement observed in the MFB samples can be primarily attributed to their lower density, slightly higher total porosity, and the presence of minerals with high specific heat capacities (Cp), such as calcite and CaSO_4_. Notably, the proportional distribution of these minerals was higher in *MFB-70* and *MFB-85*. Additionally, the Cp of carbonated *Reference*, *MFB-70,* and *MFB-85* samples were 1122.8, 1178, and 1202.6 J/(kg·K), respectively. Thermal diffusivity (α), which indicates how quickly a material responds to temperature changes, was measured as 0.25 mm^2^/s for the carbonated-*Reference*, 0.21 mm^2^/s for *MFB-70,* and 0.20 mm^2^/s for *MFB-85*. These interconnected thermal properties demonstrate the suitability of MFBs for energy-efficient and thermally stable construction applications, highlighting the importance of material selection in optimizing the insulation performance.

**Table 5:** Thermal Properties of Carbonated-*Reference*, *MFB-70,* and *MFB-85* Samples

	*k* W/(m·K)	α mm^2^ /s	Cp J/(kg·K)
*Reference*	0.21	0.25	1122.8
*MFB-70*	0.18	0.21	1178
*MFB-85*	0.17	0.20	1202.6

### Insights from TGA Analysis and Dissolution Characteristics

This section explores the TGA profiles for identification of net CO_2_ uptake values as well as hydrated phases in correlation with dissolution characteristics to estimate the formation, stability, and solubility of complex hydrated phases formed in uncarbonated and carbonated samples.

TG-DTG curves of the *Reference* and MFB specimens are shown in Figure 5. CO_2_ uptake was quantified based on the difference in mass loss attributed to the thermal decomposition of calcium carbonate (CaCO_3_) (at 575–900 °C) between uncarbonated and carbonated samples. The mass loss (marked in Figure 5a,c) from calcite decomposition is higher in MFBs due to the pre-existing calcite in OSA, compared to the *Reference* sample. The *Reference* sample showed the highest CO_2_ uptake at 14.1%, while *MFB-70* and *MFB-85* exhibited 13.7 and 12.6%, respectively.

**Figure 5:**
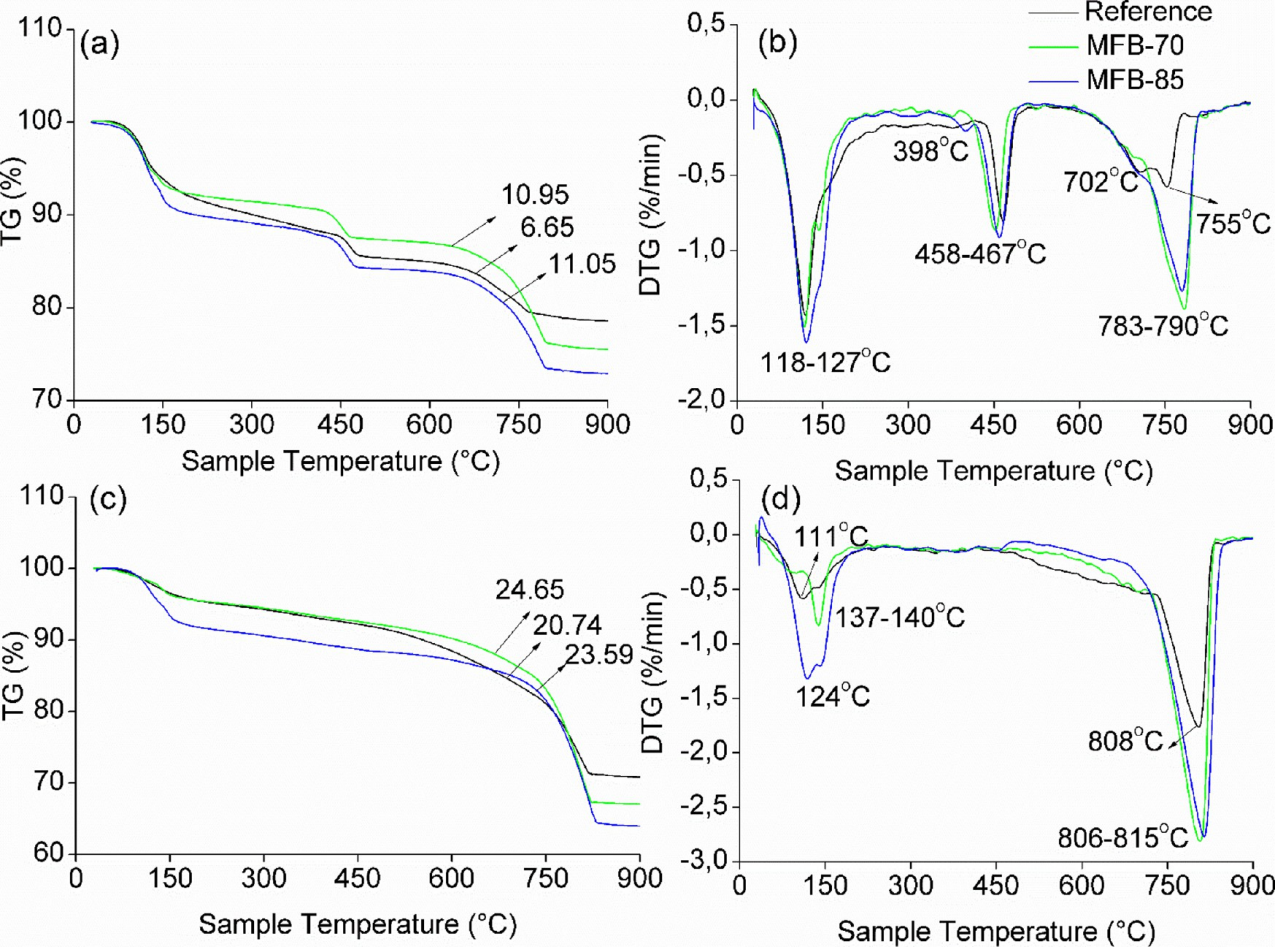
TGA-DTG curves of *Reference* and MFB with different proportions: (a) TGA and (b) DTG curves of uncarbonated samples and (c) TGA and (d) DTG curves of carbonated samples.

The initial mass loss observed in TGA is primarily attributed to the dehydration of hydration products present in both crystalline and amorphous phases, which may include calcium silicate hydrate (C–S–H), calcium aluminosilicate hydrate (C–A–S–H), calcium aluminate hydrate (C–A–H), ettringite, and gypsum.^29^ In uncarbonated samples, particularly *MFB-70* and *MFB-85*, DTG curves reveal prominent water loss peaks (118–127 °C), which are more pronounced than in the *Reference* sample. This increase in mass loss correlates with higher OSA content and is likely due to the enhanced formation of ettringite and gypsum, as confirmed by XRD analysis (Figure 6) and supported by previous findings.^29,55^ Elevated levels of OSA can increase the system pH, which may lead to the precipitation of sulfate-bearing phases such as ettringite and gypsum.^29^ This effect is particularly notable in the *MFB-85* sample, where the *pH* remains elevated (∼12.94), contributing to the stability of the hydration products prior to carbonation (Table 6).

**Table 6:** *pH/Ec* and Leaching (Ca^2+^ and SO_4_^2–^) of Uncarbonated (UC) and Carbonated (C) Samples

		*pH*	*Ec* (ms/cm)	Ca^2+^ (mg/kg)	SO_4_^2–^(mg/kg)
*Reference*	UC	12.63	9.26	1425	<5
	C	11.25	1.42	212.8	433
*MFB-70*	UC	12.75	9.68	1304.4	1750
	C	10.56	2.58	658	2150
*MFB-85*	UC	12.94	9.56	1340.3	1740
	C	11.88	2.99	766.4	2045

**Figure 6:**
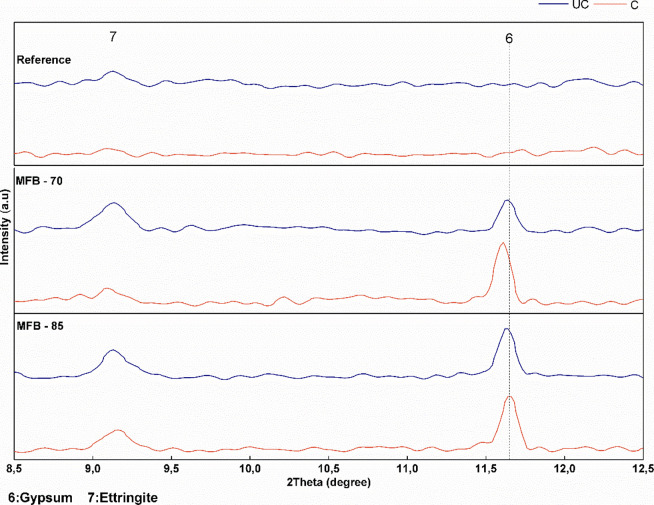
XRD spectra of uncarbonated (UC) and carbonated (C) *Reference* and MFB samples (8–13°).

After ACC treatment, all samples exhibit reduced mass loss in the 50–200 °C range, reflecting the partial transformation of C–S–H and ettringite into carbonate phases such as calcite. This is further evidenced by the emergence of a distinct DTG peak around 111 °C in the carbonated *Reference* sample (Figure 5b,d), which can be attributed to carbonation and the dihydroxylation of ettringite and gypsum (eqs 8–10).^53^ ^,^ ^56^ In addition, the DTG profile of *MFB-85* shows more intensive peaks between 137 and 140 °C, corresponding to the dehydration of gypsum­(eqs 9 and 10),^29^ which becomes more evident due to the destabilization of sulfate-bearing phases as the *pH* drops after carbonation.

The subsequent DTG stages indicate the dehydration of brucite (Mg­(OH)_2_) (Figure 5a,b) (eq 3), the decomposition of portlandite (Ca­(OH)_2_) at 458–467 °C (eq 4), and the release of CO_2_ from the decomposition of calcium carbonate (CaCO_3_) at 806–815 °C (eq 5). Carbonates decomposing below ∼750 °C are indicative of poorly crystalline phases.^53^ Nearly all portlandite is consumed after ACC treatment, indicating that carbonation effectively converts Ca­(OH)_2_—and partially C–S–H—into stable CaCO_3_ (Table 6), contributing to pore filling, reduced porosity, and lower calcium ion mobility.

$$\mathrm{Mg}{\left(\mathrm{OH}\right)}_{2}\to \mathrm{MgO}+H_{2}O$$ 3

$$\mathrm{Ca}{\left(\mathrm{OH}\right)}_{2}\to \mathrm{CaO}+H_{2}O$$ 4

$${\mathrm{CaCO}}_{3}\to \mathrm{CaO}+{\mathrm{CO}}_{2}$$ 5

$$\mathrm{Ca}{\left(\mathrm{OH}\right)}_{2}+{\mathrm{CO}}_{2}\to {\mathrm{CaCO}}_{3}+H_{2}O$$ 6

$$5\mathrm{CaO}\cdot 6{\mathrm{SiO}}_{2}\cdot 5H_{2}O+5{\mathrm{CO}}_{2}\to 5{\mathrm{CaCO}}_{3}+6{\mathrm{SiO}}_{2}+5H_{2}O$$ 7

$$3\mathrm{CaO}\cdot {\mathrm{Al}}_{2}O_{3}\cdot 3{\mathrm{CaSO}}_{4}\cdot 32H_{2}O+3{\mathrm{CO}}_{2}\to 3{\mathrm{CaCO}}_{3}+3{\mathrm{CaSO}}_{4}\cdot 2H_{2}O+{\mathrm{Al}}_{2}O_{3}+26H_{2}O$$ 8

$${\mathrm{CaSO}}_{4}\cdot 2H_{2}O\to {\mathrm{CaSO}}_{4}\cdot 1/2H_{2}O+3/2H_{2}O$$ 9

$${\mathrm{CaSO}}_{4}\cdot 1/2H_{2}O\to {\mathrm{CaSO}}_{4}+1/2H_{2}O$$ 10

Importantly, despite the partial decomposition of hydration products, *MFB-85* demonstrates a relatively lower degree of phase degradation and a less significant *pH* drop. This may indicate either a less extensive carbonation reaction or the buffering capacity of OSA, possibly due to the presence of alkali elements (Na^+^, K^+^) or pre-existing carbonate phases within the ash. These species may help stabilize *pH* and moderate carbonation kinetics, as suggested by earlier studies.^29,52^ Additionally, due to the low cement content and limited formation of calcium silicate hydrate (C–S–H), the binding of these cations within the C–S–H matrix is reduced, making them more accessible and readily dissolvable, which can contribute to an increase in pH.

The formation of a carbonated layer also decreases the availability of soluble inorganic components, as reflected in reduced *Ec* values (Table 6), while simultaneously increasing sulfate leaching due to the destabilization of ettringite and monosulfate phases at lower pH.^29^ Nevertheless, *MFB-85* and other ash-rich systems show substantial mechanical strength improvement after carbonation, likely due to carbonation-induced matrix densification and pore refinement, which are especially effective in pozzolanic-rich matrices with reactive and fine microstructures.

These findings suggest a synergistic interaction between PC and OSA, particularly at high cement replacement ratios (*MFB-85*), leading to altered hydraulic/pozzolanic activity and carbonation behavior. OSA not only enhances early hydration through interactions with sulfur-bearing phases but also influences the carbonation process by improving the chemical stability and mechanical performance of the system. This can be attributed to its pH-buffering capacity, derived from its alkali content. However, further research is needed to differentiate the complex chemical buffering effects from physical densification mechanisms and to quantitatively assess the contribution of alkalis and other buffering compounds in mitigating carbonation-induced changes in ash-rich cementitious systems.

### Effects of ACC Treatment on the Mineralogy and Morphology of MFBs

XRD and FTIR analyses were performed to identify carbonation products formed through alterations in hydrous phases within MFBs, with XRD focusing on mineralogical phase identification (Figures 6–8) and FTIR on molecular structures and chemical bonding (Figures 9 and 10).

**Figure 7:**
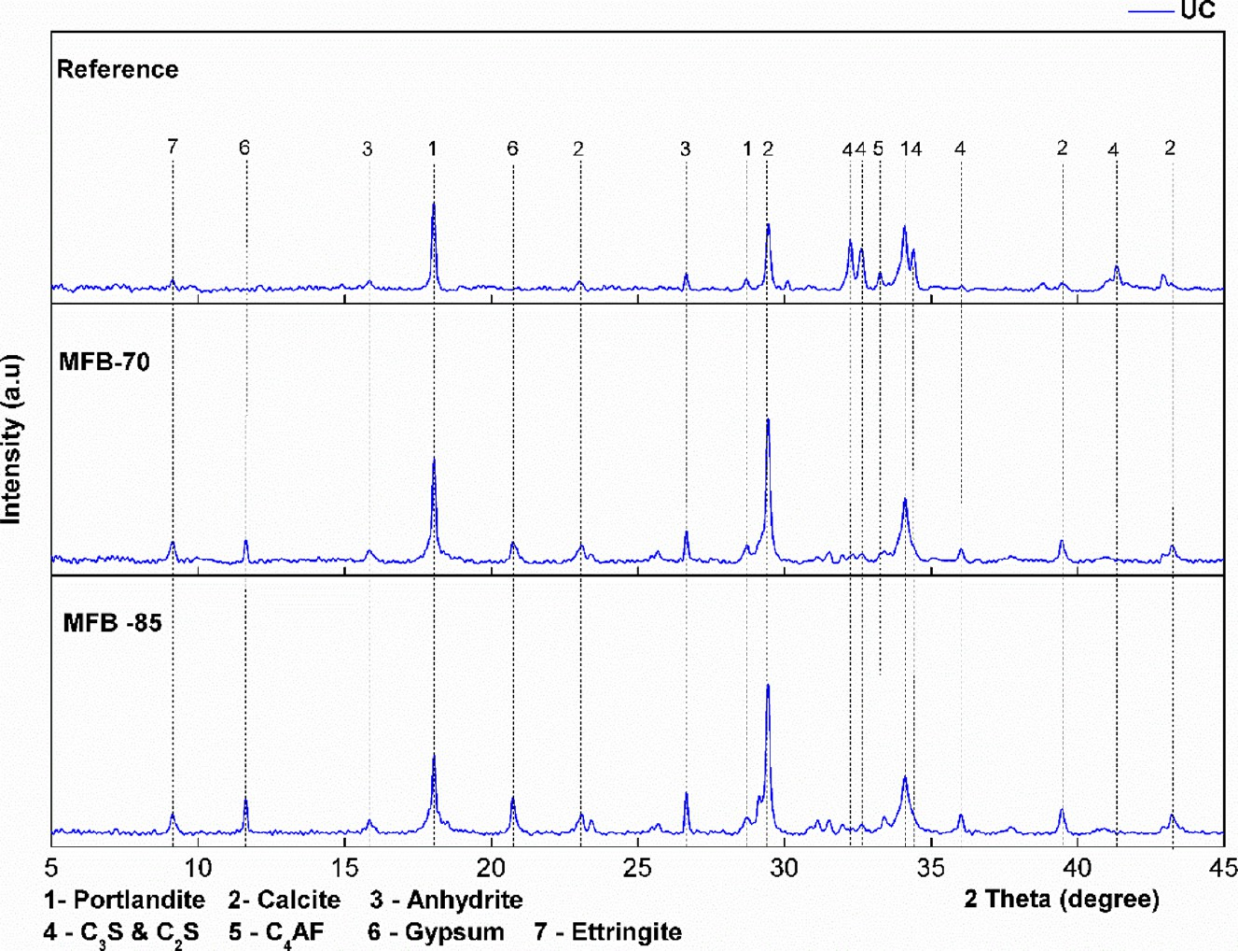
XRD spectra of uncarbonated (UC) *Reference* and MFB samples.

**Figure 8:**
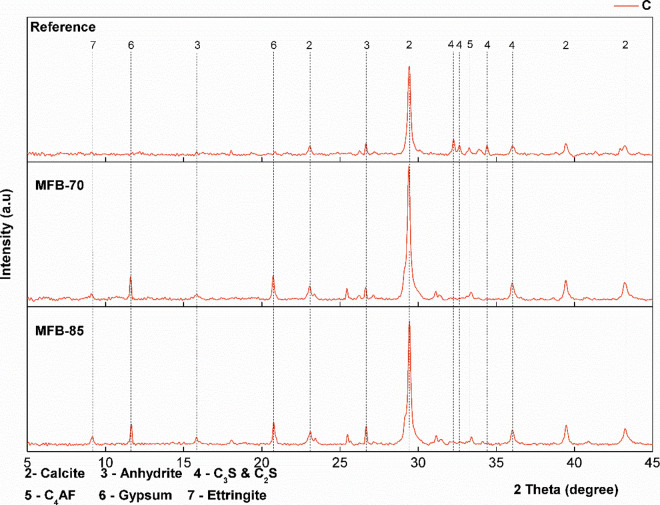
XRD spectra of carbonated (C) *Reference* and MFB samples.

**Figure 9:**
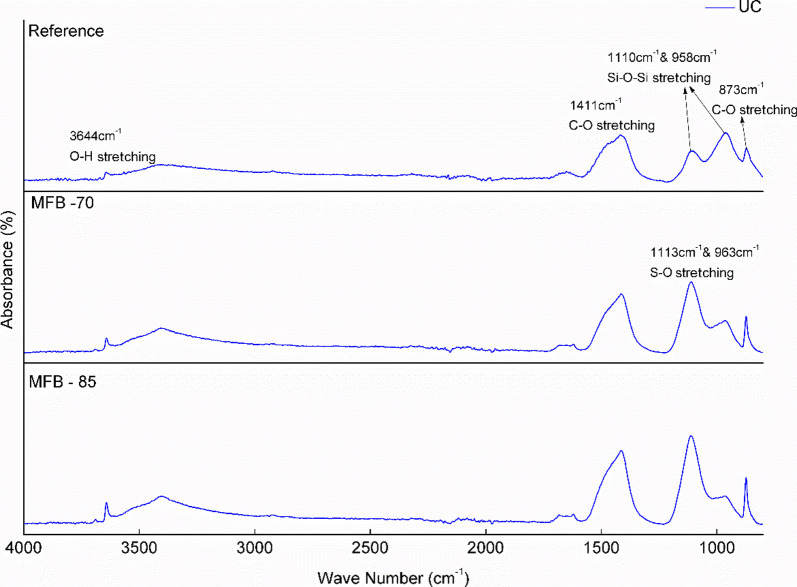
FTIR spectra of uncarbonated (UC) *Reference* and MFB samples.

**Figure 10:**
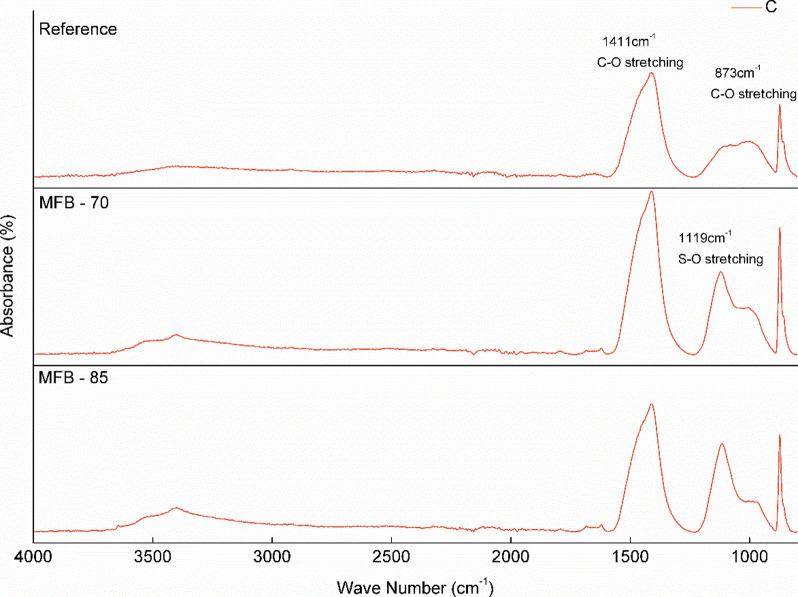
FTIR spectra of carbonated (C) *Reference* and MFB samples.

The diffraction patterns of all of the carbonated samples (*Reference* and MFBs) indicate near-total consumption of the portlandite phase, consistent with TGA findings, with calcite emerging as the dominant carbonation product, as evidenced by the intensified calcite peaks (especially at 29°) in the carbonated samples (Figures 7 and 8). FTIR analysis (Figures 9 and 10) further supports these observations, showing a reduction in the O–H stretching peaks between 3640–3644 cm^–1^, which are attributed to hydrated phases, primarily Ca­(OH)_2_, and the appearance of new phases, primarily calcite, indicated by peaks around ∼1410 to −1470 and ∼870 cm^–1^. The FTIR spectra confirm that calcite is the main polymorph of CaCO_3_ in the carbonated system, as demonstrated by the enhanced peaks at 873 cm^–1^ corresponding to the out-of-plane bending vibrations and 1411 cm^–1^, corresponding to the asymmetric stretching of carbonate ions.^56–58^

The XRD analysis also reveals a decrease in the intensity of calcium silicate and calcium aluminoferrite peaks, particularly between 30 and 35° in the reference sample. This trend becomes more pronounced with a higher ash content and ACC treatment. The observed decrease in peak intensity is attributed to an increased presence of amorphous silica gel. This interpretation is corroborated by FTIR, which also underscores the inherent difficulty in identifying poorly crystalline C–S–H phases through XRD due to their low long-range order.^56,57^ The FTIR spectra exhibit a minor shoulder at 958 cm^–1^, associated with Si–O stretching vibrations,^58,59^ and a prominent, broad absorption band with a maximum between 1110 and 1120 cm^–1^, attributed to overlapping Si–O and S–O vibrational modes in the MFB matrices.^59^ While the spectral overlap complicates definitive identification of sulfur-bearing phases, the assignment of this region to anhydrite and gypsum is more consistent with complementary XRD and TGA data (Figure 5).^57,60^

The identified phases in the carbonated MFBs include calcite (CaCO_3_), which is the main carbonation product; portlandite (Ca­(OH)_2_), which undergoes near-complete consumption during carbonation; C–S–H phases, which are partially decalcified; and sulfur-bearing phases such as anhydrite (CaSO_4_) and gypsum (CaSO_4_·2H_2_O), as evidenced by FTIR and XRD analysis. The presence of these phases reflects the complex interactions between carbonation and the materials within the MFBs (eqs 3–10), with a particular emphasis on the transformation of hydrous calcium silicates and sulfates.

The morphological evolutions were examined with SEM analysis, and the representative images of both carbonated and uncarbonated samples are presented in Figures 11–13. The uncarbonated *Reference* sample exhibits rod-shaped and needle-like ettringite formations (Figure 11b), indicative of sulfate incorporation during early hydration. Following ACC treatment, the *Reference* sample evolves into a floc-like, amorphous C–S–H that is well-connected to the surrounding matrix. This transition signifies extensive polymerization and densification of the hydrated phases, contributing to an improved mechanical performance. Concurrently, strong rhombohedral calcite crystallization occurs within micropores (Figure 11d), an equilibrium morphology commonly observed under stable thermodynamic conditions.^61^ The presence of these well-crystallized calcite formations within void spaces reinforces matrix integrity by reducing porosity and enhancing compressive strength through improved particle packing.^17^

**Figure 11:**
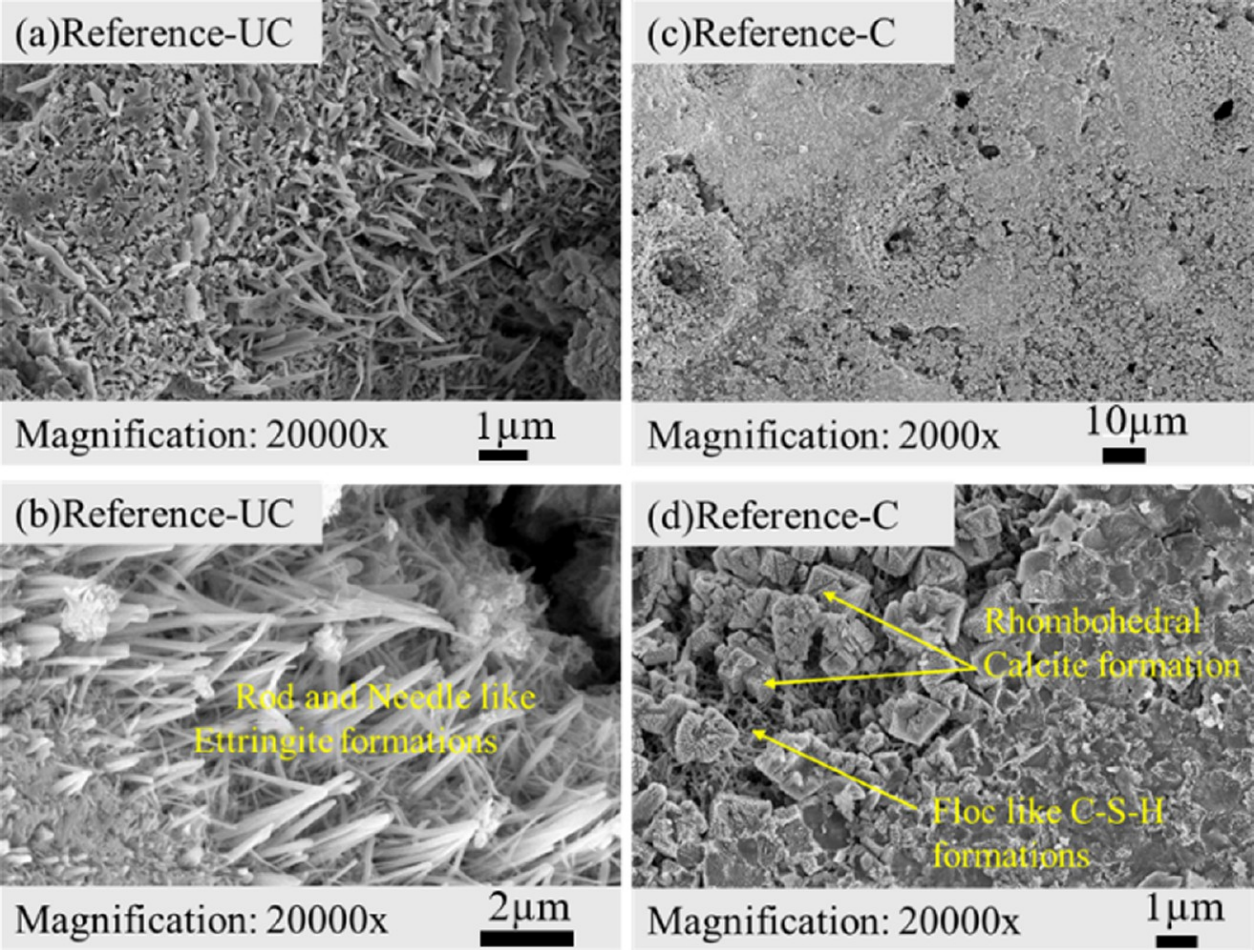
SEM images of (a, b) uncarbonated (UC) and (c, d) carbonated (C) *Reference* sample.

**Figure 12:**
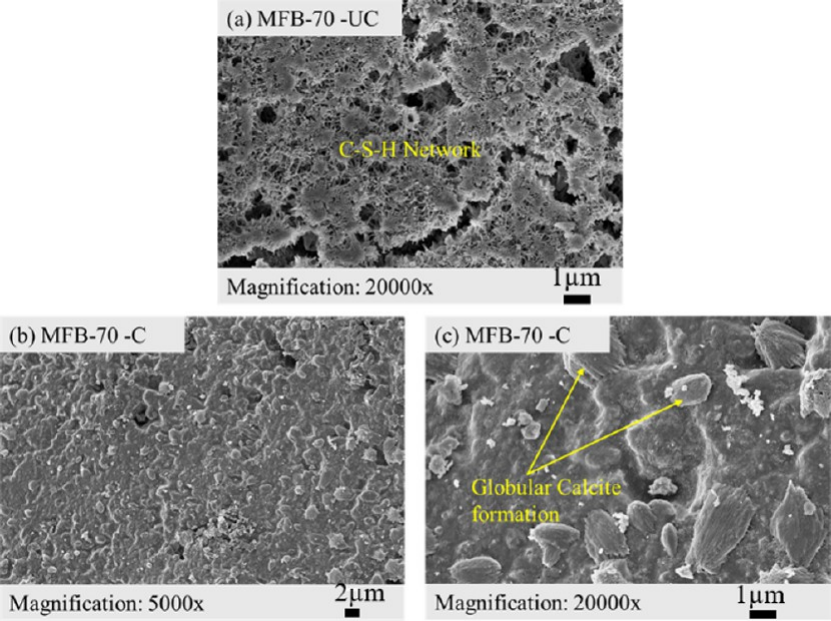
SEM images of (a) uncarbonated (UC) and (b, c) carbonated (C) *MFB-70* sample.

**Figure 13:**
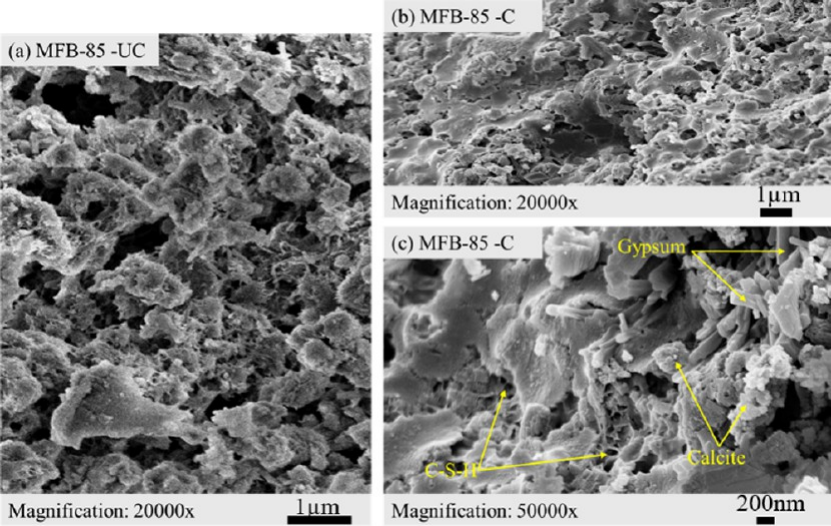
SEM images of (a) uncarbonated (UC) and (b, c) carbonated (C) *MFB-85* sample.

The microstructure of the *MFB-70* sample shows C–S–H morphology similar to that of the *Reference* sample; however, it does not display rhombohedral calcite formations. Instead, globular, flower-like calcite nucleation is visible (Figure 12c). As the ash content increases, the C–S–H phase becomes more heterogeneous, with a transition occurring in the pore structure, leading to a less interconnected matrix and varying pore sizes. As a result, the sample exhibits a highly heterogeneous microstructure, characterized by poorly connected, floc-like amorphous C–S–H and more dispersed nonuniform globular calcite morphologies (Figure 13b,c). Gypsum is also better detected in the carbonated *MFB-85* sample compared to *MFB-70*, as evidenced by SEM imaging (Figure 13c) and confirmed through XRD analysis (Figure 8). The presence of gypsum can trigger sulfate interactions within the system, potentially influencing the microstructural evolution of the *MFB-85* sample.^62^

## Conclusions

This study examines the impact of ACC treatment on the performance of the MFBs. The findings emphasize the role of ACC treatment in enhancing structural performance, while promoting environmental sustainability.

The primary conclusions derived from the study are as follows:

- OSA incorporation can offer advantages in thermal properties of MFBs, yet without ACC treatment, the strength development of MFBs was primarily governed by cement hydration. From a mechanical performance perspective, the incorporation of OSA into cement increases water demand, potentially reducing mechanical strength.
- Even so, the interaction between hydration phases and CO_2_ mineralization (∼140 kg/ton) during ACC treatment facilitates beneficial secondary reactions, which significantly enhance the material’s structural integrity and result in a substantial increase in compressive strength values.
- Carbonated samples exhibited reduced total porosities as a result of CaCO_3_ formation, which was also demonstrated by FTIR and XRD, altering microstructures and morphology favorably with the pore-filling effect of calcite crystals.
- ACC treatment significantly influences the microstructure of OSA-blended cementitious materials. Microstructural analysis reveals that *MFB-70* resembles the uncarbonated *Reference* sample, with abundant C–S–H and calcite crystals, whereas *MFB-85* shows fewer C–S–H phases and dispersed globular calcite.
- TGA patterns reveal clear changes in hydrated phases (e.g., C–S–H, ettringite) with increasing OSA content. In *MFB-85*, this leads to more hydrous phases overall—reduced C–S–H and increased ettringite and gypsum due to alkali buffering. Further research is needed to separate chemical buffering from physical densification and to quantify the role of alkalis in carbonation resistance.

These findings highlight that ACC-treated MFBs enable greater incorporation of OSA into cement-based materials beyond conventional limits. This **demonstrates** the potential of utilizing calcium- and sulfur-rich byproducts in lightweight construction materials that balance mechanical strength, thermal performance, and CO_2_ sequestration.
